# Artificial light on water attracts turtle hatchlings during their near shore transit

**DOI:** 10.1098/rsos.160142

**Published:** 2016-05-18

**Authors:** Michele Thums, Scott D. Whiting, Julia Reisser, Kellie L. Pendoley, Charitha B. Pattiaratchi, Maira Proietti, Yasha Hetzel, Rebecca Fisher, Mark G. Meekan

**Affiliations:** 1 Australian Institute of Marine Science c/o The UWA Oceans Institute (MO96), University of Western Australia, 35 Stirling Highway, Crawley, Western Australia 6009, Australia; 2 Indian Ocean Marine Research Centre and UWA Oceans Institute, University of Western Australia, 35 Stirling Highway, Crawley, Western Australia 6009, Australia; 3 School of Civil, Environmental, and Mining Engineering (M015), University of Western Australia, 35 Stirling Highway, Crawley, Western Australia 6009, Australia; 4 Marine Science Program, Department of Parks and Wildlife, 17 Dick Perry Avenue, Kensington, Western Australia 6151, Australia; 5 The Ocean Cleanup Foundation, Torenhove, Martinus Nijhofflaan 2, 18th floor, Delft 2624 ES, The Netherlands; 6 Pendoley Environmental Pty Ltd, 2/1 Aldous Place, Booragoon, Western Australia 6154, Australia; 7 Instituto de Oceanografia, Universidade Federal do Rio Grande, Avenida Italia km 08, Rio Grande, Rio Grande do Sul 96203-900, Brazil

**Keywords:** acoustic telemetry, in-water movement, VR2W positioning system, green turtle, light pollution, coastal development

## Abstract

We examined the effect of artificial light on the near shore trajectories of turtle hatchlings dispersing from natal beaches. Green turtle (*Chelonia mydas*) hatchlings were tagged with miniature acoustic transmitters and their movements tracked within an underwater array of 36 acoustic receivers placed in the near shore zone. A total of 40 hatchlings were tracked, 20 of which were subjected to artificial light during their transit of the array. At the same time, we measured current speed and direction, which were highly variable within and between experimental nights and treatments. Artificial lighting affected hatchling behaviour, with 88% of individual trajectories oriented towards the light and spending, on average, 23% more time in the 2.25 ha tracking array (19.5 ± 5 min) than under ambient light conditions (15.8 ± 5 min). Current speed had little to no effect on the bearing (angular direction) of the hatchling tracks when artificial light was present, but under ambient conditions it influenced the bearing of the tracks when current direction was offshore and above speeds of approximately 32.5 cm s^−1^. This is the first experimental evidence that wild turtle hatchlings are attracted to artificial light after entering the ocean, a behaviour that is likely to subject them to greater risk of predation. The experimental protocol described in this study can be used to assess the effect of anthropogenic (light pollution, noise, etc.) and natural (wave action, current, wind, moonlight) influences on the in-water movements of sea turtle hatchlings during the early phase of dispersal.

## 1. Introduction

The widespread use of artificial light in modern societies means that light pollution is an increasingly common feature of the environments humans inhabit. This type of pollution is exceptionally high in coastal regions of tropic and temperate zones, as these are areas of high rates of human population growth and settlement. Light pollution is a threat for many species that inhabit these locations, particularly those whose ecology or behaviour depends, in some way, on natural cycles of light and dark [1].

Artificial light is known to have detrimental effects on the ecology of sea turtles, particularly at the hatchling stage when they emerge from nests on natal beaches and head towards the sea [2–4]. Under natural conditions, turtles hatch predominantly at night (although some early morning and late afternoon emergences occur) and show an innate and well-directed orientation to the water, relying mostly on light cues that attract them toward the brighter horizon above the sea surface [4–6]. Artificial lighting on beaches is strongly attractive to hatchlings and can cause them to move away from the sea and interfere with their ability to orient in a constant direction [4]. Ultimately, this disorientation due to light pollution can lead to death of hatchlings from exhaustion, dehydration and predation [4].

While the problems caused by light pollution during the journey of hatchlings from the nest to the water's edge are well recognized, the impact of artificial light on their behaviour once they reach the water is unknown. Upon arrival at the sea, turtle hatchlings swim to offshore waters to begin their oceanic life stage, orientating using wave direction [7] and an internal magnetic compass [8,9]. Laboratory experiments have been inconclusive as to which cue is most influential on hatchling orientation, but artificial light has been shown to affect their in-water swimming behaviour [10,11]. There are also many anecdotal observations of wild hatchlings swimming around lights at the sea surface (see [4] for a review), suggesting that light continues to be an important navigational cue once hatchlings enter the water.

The near shore environment is host to many animals (e.g. reef fishes, sharks) known to predate turtle hatchlings. Predation risk is greatest close to shore in shallow water [12], and highest mortality occurs over the first hour after entering the sea [13]. Therefore, hatchlings transit quickly through this hostile environment to maximize their chances of survival in a period known as the ‘frenzy’ that is characterized by almost continuous swimming for up to 24 h [14]. If light pollution disrupts the orientation and swimming behaviour of hatchlings causing them to linger or become disoriented in the near shore, it is likely to increase the chances of mortality, with detrimental effects on the survivorship and resilience of populations.

The effects of artificial light on the in-water movement of newly hatched turtles is still unknown, since until very recently we lacked the technology to follow hatchlings in near shore waters at night. Thums *et al.* [15] demonstrated that this was in fact possible, using new miniature acoustic transmitters and passive receiver arrays, allowing multiple individuals to be tracked simultaneously within a narrow time-window (a few hours).

Here, we used the approach of Thums *et al.* [15] to examine the effect of artificial light on the behaviour of green turtle (*Chelonia mydas*) hatchlings as they entered the sea from a remote beach at North West Cape, Ningaloo Reef, Western Australia. We tracked turtle hatchlings in near shore waters under conditions of ambient and artificial light and assessed the effect of artificial light on swimming direction and behaviour.

## 2. Material and methods

### 2.1. Study site

**Figure 1.**
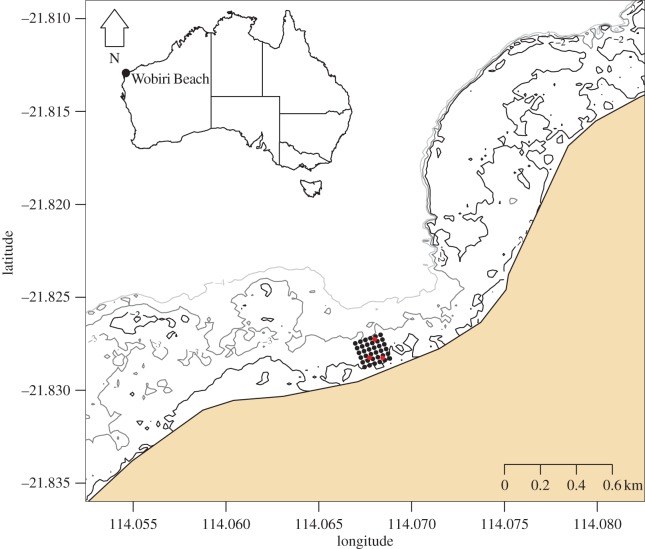
Map of the study site at Wobiri Beach, North West Cape, Western Australia and inset map of Australia showing the position of the study site. Map shows acoustic receivers in black and reference tags in red. Three bathymetry contours are shown: 2 m in black, 3 m in dark grey and 4 m in light grey.

We tracked green turtle (*C. mydas*) hatchlings at night in March 2014 at Wobiri Beach (21°49′42.49^″^S, 114° 4′5.99^″^E), North West Cape, Western Australia (figure 1). This beach is subject to low to medium energy swell conditions (1–2 m). Our experiments were conducted around midnight (table 1), after moon set on a rising tide. High tide occurred at 01.09 h on the first night (2.54 m) and at 02.39 h on the second night (2.26 m), as estimated by the Australian Bureau of Meteorology tide chart for Exmouth.

**Table 1.** Details of each of the 10 turtle hatchlings released into the tracking array in each treatment in each night. SCL, straight carapace length; SCW, straight carapace width. Time indicates when the first pair of turtles were released for each treatment.

treatment	date	time	nest	hatch date	SCL (mm)	SCW (mm)	mass (g)
ambient	5 Mar	23.05	1	4 Mar	48.3 ± 0.7	38.2 ± 1.0	25.6 ± 0.9
light	5 Mar	00.05	1	4 Mar	48.7 ± 1.4	37.6 ± 1.3	26.1 ± 1.0
mean					48.5 ± 1.1	37.9 ± 1.1	25.9 ± 1.0
ambient	7 Mar	01.00	^a^2, 3	6, 7 Mar	47.4 ± 1.7	37.9 ± 2.8	22.8 ± 2.8
light	7 Mar	23.25	^b^2, 3	6, 7 Mar	47.2 ± 1.6	37.8 ± 1.7	21.9 ± 2.9
mean					47.3 ± 1.6	37.9 ± 2.3	22.4 ± 2.8

a Three of these turtles were from nest 2 and the remainder from nest 3.

b One turtle was from nest 2 and the remainder from nest 3.

### 2.2. Animal capture and handling

Hatchlings were removed from three nests at the time of emergence in the early hours of the morning, held in an insulated box with a moist sand floor and kept in a cool, dark room until the moon set of the following night, when they were released as part of the experiment. We attempted to obtain hatchlings from one nest for each night of the experiments; however, on the second night there were insufficient hatchlings from one nest, thus hatchlings from two nests were used (table 1). We measured straight carapace length and width (±0.01 cm) and body mass of each hatchling using digital calipers and scales (±0.1 g; table 1).

### 2.3. Transmitter deployment

**Figure 2.**
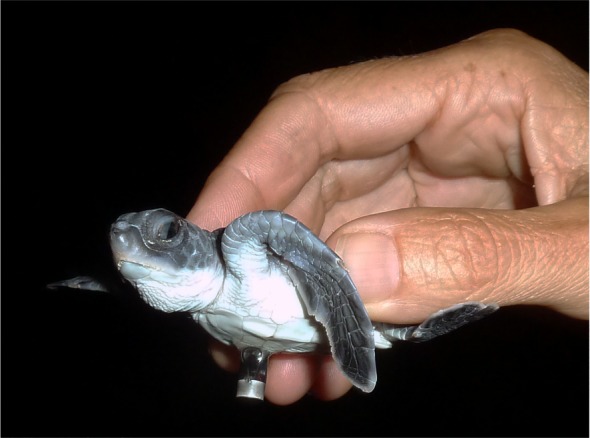
A green turtle hatchling with the Vemco V5 acoustic transmitter attached.

We tracked 40 hatchlings using V5 180 kHz coded acoustic transmitters (Vemco Ltd, Halifax, Canada) programmed with a delay of 5–10 s. These were the smallest acoustic tracking devices available (mass 0.65 g, length 12 mm, diameter 5 mm). Transmitters weighed approximately 3% of the average body mass of green turtle hatchlings, below the threshold where negative effects of the tag on locomotion are thought to occur [16–18]. Transmitters were glued to the turtle's underside with the transducer pointing down using a small drop of fast-acting epoxy (figure 2). This orientation of the tag would have increased the drag experienced by hatchlings [19] compared with previous hatchling tracking studies where tags were oriented horizontally on hatchlings [15,20]. However, any drag effects were consistent across treatments and our pilot study showed that a vertical orientation of the transmitter was necessary for the successful functioning of the positioning system (at least three receivers must hear each transmission for a successful position to be calculated; see electronic supplementary material, S1 and table S1). The transmitters were glued to the hatchlings approximately 1–2 h prior to release. Based on previous studies, we estimated that tags would detach from each animal after one to two weeks [21].

### 2.4. Acoustic tracking array

**Figure 3.**
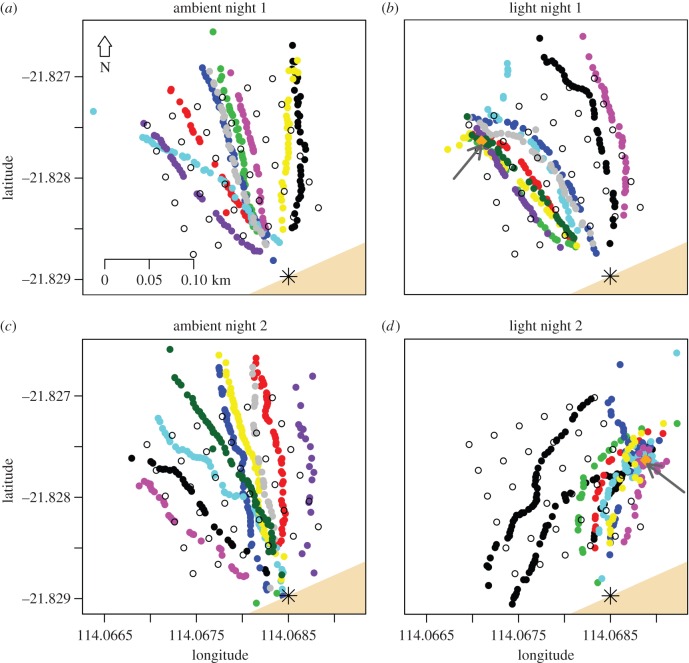
Tracks from each individual hatchling shown in different colours. Open circles represent the positions of each of the receivers in the tracking array and the beach is shown in beige at the bottom of the plots. The asterisk indicates the release site of turtles at the water's edge. (*a*,*c*) The tracks of turtles in the ambient treatments, with no light present and (*b*,*d*) the tracks of turtles in the light treatments with the position of the light indicated by the orange filled diamonds and dark grey arrows.

We deployed a 6 × 6 array of 36 VR2W acoustic receivers (Vemco Ltd, Halifax, Canada) in parallel rows of six beginning 30 m from the low tide mark, with each row separated by 30 m and each receiver within a row separated from the nearest neighbour by a minimum of 30 m (figure 3). Distance between receivers was determined by a pilot study and the manufacturer's recommendations (see the electronic supplementary material). Receivers were attached to a 1.5 m mooring line (4 mm polypropylene rope) held in position with a 100 mm subsurface float and a 3 kg weight. A synchronizing transmitter (allows the synching of time logged by each receiver) was attached approximately 0.5 m above each receiver and 0.5 m below the surface of the water. A current meter (Nortek AS Aquadopp) was also deployed near the centre of the array in 2–3 m of water. The current meter sampled for 3 min every 15 min and averaged those observations, resulting in current speed and direction measurement every 18 min.

### 2.5. Light experiment

We released tagged hatchlings into the array under conditions of both ambient and artificial light over two nights (table 1). All turtles were released into the array from the water's edge, aligned near to the middle of the array (figure 3). Turtles were held at the water surface for approximately 30 seconds prior to release. Artificial lighting was provided by a 400 W metal-halide light powered by a generator. The light was deployed on an 8.25 m boat moored at the edge of the array with the light facing the beach, creating a light loom on the water, to one side of the boat. On the first night, five pairs of tagged turtles were released into the array. A pair was released every 10 min following moon set under ambient light conditions, which included a low light-glow visible on the horizon from the small town of Exmouth (12 km southeast). An hour later, the artificial light was illuminated and another five pairs of tagged turtles were released into the array at 10 min intervals. The light was switched off 90 min later. Pairs of tagged turtles were released at 10 min intervals in order to reduce potential collisions of acoustic signals from multiple transmitters [22]. On the following night, the order of light treatments was reversed. We also switched the position of the light to the opposite side of the array to account for any potentially confounding effects of current flow.

We quantified light in the environment using a Canon PowerShot G12 camera with an unfiltered Raynox DCR-CF187PRO HD fish eye lens. The fish eye lens enables the entire night sky and horizon to be captured in a single circular image. The camera was deployed on the beach near the turtle release point and photos of the night sky were taken at 15 min intervals during the experiments. We then made isophote images of the photographic data, by extracting the short wavelength violet and blue light most relevant to marine turtle hatchlings (450–500 nm) [23–25]. White lights enriched in short wavelength violet and blue light include white LED, metal halide, mercury vapour and fluorescent.

### 2.6. Data analysis

The detection data were processed by Vemco Ltd to generate position estimates of turtles within the array, so that turtle tracks could be reconstructed. For each of the turtle tracks we calculated the total time detected by receivers in the array, the speed of each turtle (distance between each point divided by the time taken to travel that distance) and the bearing (relative to 0° such that westerly bearings would be negative) from the release point to the point where it left the tracking array (electronic supplementary material, table S2). We then associated the closest current speed and direction measurement with the timing of each turtle's transit (electronic supplementary material, table S2). For each turtle's transit there was typically one measurement of current speed and direction (78% of turtles), with 8% of turtles having two measurements, and for 14% of turtles their transit did not correspond with any measurements of current speed and direction. For these five turtles we used the closest current measurement in time (electronic supplementary material, table S2). We then used time spent in the tracking array, speed and bearing as response variables in a suite of generalized additive models with treatment (artificial versus ambient), night of experiment, current speed and current direction as the predictor variables. We used the gam function in the mgcv library [26] in R [27] to fit models consisting of all combinations of the predictor variables. All response variables were fitted with a Gaussian distribution. Note that bearing data were normally distributed as turtles remained within the northeast and northwest compass quadrant (range = −48°–13°), thus making it possible to model the data adequately using a Gaussian distribution. Current speed was modelled with a cubic regression spline and current direction modelled with a cyclic cubic regression spline, i.e. a penalized cubic regression splines whose ends match, up to second derivative. As the latter smoother includes shrinkage by default, the shrinkage version of the cubic regression spline was also implemented for current speed. We restricted the basis dimension *k* < 5 to avoid overfitting, and calculated the number of parameters in each model as the number of fixed terms plus the number of fitted lines. The models were compared and ranked according to Akaike's information criterion, corrected for small sample size (AIC_c_) and by their relative goodness of fit, the AIC_c_ weight. The AIC_c_ weight varies from 0 (no support) to 1 (complete support) [28]. The amount of variance (percentage deviance) in the response variable explained by each of the candidate models was used as a measure of goodness-of-fit to the data [28].

We used a similar statistical procedure (full subsets modelled and ranked according to AIC_c_) to assess any differences in turtle mass between treatment and night. As these models contained only factor predictor variables, they were fitted using generalized linear models (function glm, stats package) (R [27]).

### 2.7. Transmitter effect test

In order to test if transmitters affected hatchling swimming, in the morning after the light experiments we released and followed 10 individual turtles near the shore using a kayak. Five of these turtles were fitted with transmitters, which were attached horizontally to the long axis of the turtle to allow easy removal at the end of the tracking period. Transmitters were orientated horizontally for these tests as less glue was required. This was due to the very small surface area of the rounded distal ends of the transmitter, meaning that a blob of thick glue was needed to ensure attachment in the vertical position, whereas in the horizontal position the larger surface area ensured only a small spot of thin glue was sufficient. Each turtle was followed for 10 min and the surfacing rate was recorded as an indicator of oxygen consumption and swimming effort [29]. The kayak's track was recorded using a GPS. To analyse this dataset, we used linear mixed-effects models from the nlme library in R [30] where individual turtles were the random effect, and tested for an effect of the tag by comparing the AIC_c_ and model weights of the slope model (surfacing rate ∼ tag treatment + random effect) to the intercept-only (null) model (surfacing rate ∼ 1 + random effect).

## 3. Results

For the models examining difference in turtle mass, the model with night only had the highest support, *w*AIC_c_ = 0.70 (with only 21% support for the model including treatment and 9% support for the interaction between the two), showing that although turtles released on the first night were slightly larger than on the second night, turtles were of similar sizes between treatments on each night of the experiment (table 1).

### 3.1. Acoustic array

A total of 14 806 transmissions from tags deployed on turtles were received and 65% of these were detected on at least three receivers, 54% could be used to calculate position estimates, and on average five receivers recorded each tag transmission. Two turtles (7624 and 7598) had a very low number of detections (approx. 6% of the mean (324 ± 60)). It was possible to calculate the time spent in the array with this detection data, however positions could not be estimated and, therefore, bearing and speed could not be calculated for these two turtles. On the second night, two transmitters were not activated. One hatchling returned to shore after arriving at the light and then finally headed away from shore a second time and left the array (black track in figure 3*d*). This turtle was removed from the analysis of time spent in the array. Another hatchling displayed an aberrant track and remained in the array for 28.36 h (electronic supplementary material, figure S2). This latter turtle was removed from all analyses.

### 3.2. Light experiment

Cameras revealed low light levels, typical of rural skies, over both nights of the experiment, prior to the illumination of artificial lights (electronic supplementary material, figure S1). When artificial lights were switched on, the camera measured high uni-directional light levels, consistent with anthropogenic sources in high-density urban environments (electronic supplementary material, figure S1).

**Figure 4.**
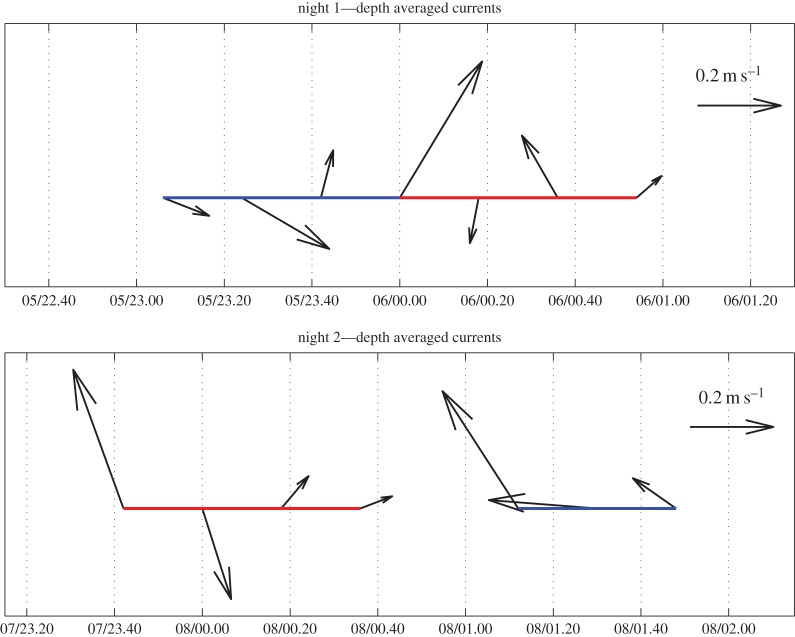
Stick plots showing the current direction and speed (length of the arrow) in each of the treatments, with light treatments in red and ambient treatments in blue.

Wind and wave conditions differed over the two nights of experiments. On the first night a strong wind blowing from the west–northwest generated small wind waves that broke on the shore with an incidence angle of approximately 45°, disrupting the swell waves that usually break parallel to the shoreline. Winds were negligible on the second night and only very small swell waves (less than 0.3 m) broke parallel to the shore. Current direction was extremely variable within and between experimental trials, except during the ambient light treatment on the second night, when it flowed consistently towards the northwest quadrant (figure 4). Current speed was also highly variable (figure 4). The strong winds on the first night combined with shallow water resulted in the position of the boat (with the light) being displaced further along the left side of the array than planned (the middle was planned, as was achieved on night 2). On the first night, the light was positioned 319° (northwest) from the release point on the shore and on the second night 12° (approx. north-northeast) from the release point.

**Figure 5.**
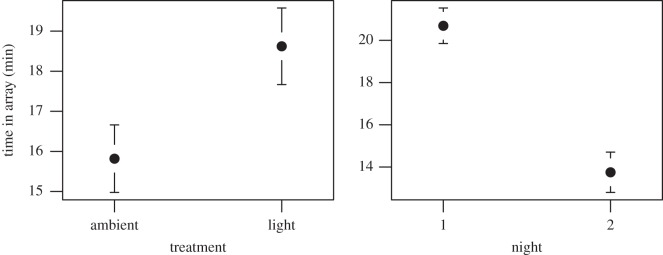
Predicted values (mean and standard error) from the model used to explain the relationship between the time turtle hatchlings spent in the tracking array for each of the treatments (ambient and light) and nights of the experiment.

Our analysis of the time hatchlings spent in the array showed that the top nine models were all within two AIC_c_ points. Thus the most parsimonious model was the one with the least number of parameters, that included only light treatment and night of experiment, which explained 54% of the deviance (table 2). Turtles spent a longer time (23%) in the array in the light (19.49 ± 5.12 min) than in the ambient treatments (15.82 ± 5.11 min) and spent longer (55%) in the array on the first night (20.69 ± 3.64 min) than on the second night (13.34 ± 4.35 min) (figure 5).

**Table 2.** Ranked (by AIC_c_) additive models of time spent in array (time) explained by light treatment (treat), current speed (CS) and direction (CD) and night of the experiment. Shown are the maximum log-likelihood (LL), the number of fitted lines (mod size), Akaike's information criterion corrected for small samples (AIC_c_), change in AIC_c_ relative to the top-ranked model (ΔAIC_c_), AIC_c_ weights (*w*AIC_c_) and the percentage deviance explained (%DE). Only model outputs within two AIC_c_ points are shown.

	model	LL	mod size	AIC_c_	ΔAIC_c_	*w*AIC_c_	%DE
	∼ night + treat	−97.27	3	203.82	0.00	0.09	0.54
	∼ CD × night + treat + night	−97.27	5	203.82	0.00	0.09	0.54
	∼ CD × treat + treat + night	−97.27	5	203.82	0.00	0.09	0.54
	∼ CS × night + treat + night	−96.94	5	204.15	0.33	0.08	0.54
	∼ CS × night + treat + night + CD × night + treat + night	−96.94	7	204.15	0.33	0.08	0.54
	∼ CS × treat + treat + night	−96.47	5	204.42	0.60	0.07	0.56
	∼ CS × treat + treat + night + CD × treat + treat + night	−96.47	7	204.42	0.60	0.07	0.56
	∼ CS × treat × night + treat × night	−94.94	8	204.62	0.80	0.06	0.59
	∼ CS × treat × night + treat × night + CD × treat × night + treat × night	−94.94	12	204.62	0.80	0.06	0.59
	∼ night	−99.74	2	206.22	2.40	0.03	0.47

We found no evidence for an effect of light treatment on average speed (49.4 ± 7.6 cm s^−1^) of hatchlings within the array (the NULL model was ranked highest).

**Figure 6.**
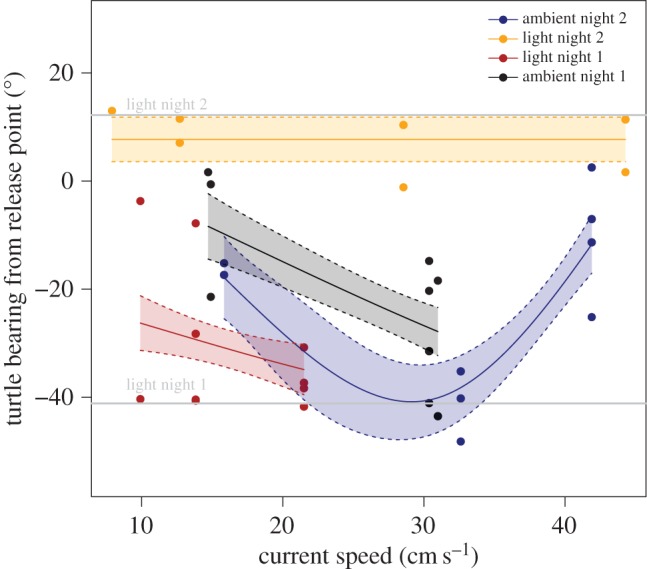
Relationship between turtle hatchling bearing from the release point (relative to 0°) and the independent variables in the top model; the interaction between current direction and treatment (table 3). The predicted smoothers are shown as solid lines and the dashed lines and transparent polygons show the standard error.

Despite the differing conditions, the tracks of the hatchlings in the ambient light treatments fanned out from the beach in a similar manner on both nights (figure 3*a,c*). In contrast, the tracks in the light treatments showed that hatchlings were strongly attracted to the light (figure 3*b*,*d*). On the first night all hatchlings except two (8/10) swam to the light, and on the second night all the turtles went towards the light (6/6) (figure 3*b*,*d*). Of the models describing the bearing of the hatchling tracks from the release point, two models had equal statistical support, the first including the three-way interaction between current speed, light treatment and night of experiment (table 3 and figure 6), and the second including both the three-way interaction between current speed, light and night and the three-way interaction between current direction, light and night (table 3; electronic supplementary material, figure S3). However, the former was the most parsimonious (8 parameters compared with 12) and explained 72% of the deviance (table 3). There was a similar weak relationship between turtle bearing and current speed for both treatments on the first night (figure 6), with turtle bearings becoming slightly more westerly as current speed increased. On the second night, there was no relationship between turtle bearing and current speed in the light treatment, however in the ambient light treatment the relationship switched from negative to positive at current speeds greater than 32.5 cm s^−1^. This was possibly due to the combination of high current speeds and current direction consistently in the same quadrant (northwest; electronic supplementary material, table S2) as the bearing taken by turtles in this treatment (figures 4 and 6). While two turtles (7609 and 7597; electronic supplementary material, table S2) in the light treatment were also subject to current speeds greater than 32.5 cm s^−1^ (figure 6) with similar current direction (−20°; electronic supplementary material, table S2), their bearing appeared to be more influenced by light (figure 6). Thus there was no clear, consistent relationship between bearings of turtles and current flow and current bearing. In both light treatments, the turtle bearing was closer to the light bearing (−41° and 12°, respectively) than in the ambient light treatments.

**Table 3.** Ranked additive models (top six models are shown) of bearing taken by hatchlings explained by current speed (CS) and direction (CD), light treatment (treat) and night of the experiment. See caption of table 2 for a description of other elements.

	model	LL	mod size	AIC_c_	ΔAIC_c_	*w*AIC_c_	%DE
	∼ CS × treat × night + treat × night	−130.52	8	282.42	0	0.35	0.72
	∼ CS × treat × night + treat × night + CD × treat × night + treat × night	−130.52	12	282.42	0.006	0.35	0.72
	∼ CS × treat + treat + night + CD × treat + treat + night	−131.54	7	285.09	2.68	0.09	0.70
	∼ CD × treat × night + treat × night	−134.78	8	285.13	2.71	0.09	0.64
	∼ CS + treat × night	−133.16	5	286.99	4.58	0.04	0.67
	∼ CS + treat × night + CD + treat × night	−133.16	6	286.99	4.58	0.04	0.67

### 3.3. Transmitter effect test

There was no evidence for a difference in rate of surfacing of tagged (horizontally aligned) and untagged hatchlings, with the null model having the highest support (*w*AIC_c_ = 0.70). Surfacing rates were 8.5 ± 6.2 s for the tagged and 8.5 ± 4.2 s for the untagged hatchlings, and turtles in each group had similar body mass (23.4 ± 0.9 g for tagged and 23.1 ± 1.2 g for untagged).

## 4. Discussion

Our study provides the first experimental evidence that wild sea turtle hatchlings can be strongly attracted to artificial light during their near shore transit. Such positive phototaxis caused swimming hatchlings to linger in the near shore zone, potentially increasing their risk of predation [13]. Our results have important implications for understanding the extent of impacts caused by changes in natural light environments near turtle rookeries.

In both experimental trials under ambient light conditions, hatchling trajectories fanned out in a similar manner across the array, mostly oriented towards north to north northwest from the point of release. In contrast, during the artificial light treatments, 80% (first night) and 100% (second night) of turtle trajectories oriented to the vicinity of the light. Current speed and direction were highly variable, probably leading to the different relationships we found between turtle bearing and current speed. We found no relationship with current direction in the models. However, there was an indication that the combined effects of high current speed and direction had an effect on turtle behaviour under ambient conditions on the second night. While it is likely that relationships between turtle behaviour and current speed are also influenced by current direction, modelling these concurrently in our data was not possible due to the relatively large sample sizes (across a broad range of both predictors) generally required to reliably fit generalized additive models. Ideally, tensor splines that allow bivariate smoothers across both predictors could be used, although these are not yet widely available in cyclic form (as required for current direction). What was clear from the modelling was that turtle bearing was more closely aligned with the position of the lights regardless of current speed (and direction), which were highly variable. This was perhaps as expected, given that on entering the ocean turtle hatchlings swim actively for a period of hours to days, after which they become more passive swimmers and disperse with ocean currents [14,20].

Light also appeared to influence hatchling movements more than wave direction in both light treatments; however, this was most obvious on the second night, where the angle to the light had a greater offset to the turtle trajectories in the ambient light treatments. Even in the ambient light treatments the bearing taken by turtles was similar, despite differences in wave conditions, with a wind wave approaching at 45° to the shoreline on the first night and a very small swell (refracted) wave approaching parallel to the shoreline on the second night. However, wind-generated waves might not elicit the same behavioural as refracted waves as is the case in loggerhead turtle (*Caretta caretta*) hatchlings [31]. In addition, the turtle hatchling trajectories we show are a combination of turtle swimming and current, and we were not able to explicitly measure turtle heading direction as animals were passively tracked. The turtle that swam to the light and then back to shore was clearly disorientated, however after it left the shore for the second time it did not swim towards the light, but left the array in a north-northeasterly direction.

We found no evidence that the speed of hatchlings was affected by artificial light (or current speed and direction), suggesting that light only influenced orientation rather than swimming speeds. However, a model allowing an interaction between current speed and direction might show a stronger effect of these oceanographic variables on swimming speed. We also found no evidence for an effect of the transmitter on hatchling surfacing rate. Although we did not adequately account for drag in our transmitter test, even if drag effects were present, they were consistent across treatments. While this is not a conclusive test of the effect of the tag on our calculated rates of travel, we suggest that any effects over the short spatial and temporal scales of our trials would be minimal. We foresee that as the use of these transmitters on turtle hatchlings increases [15,20], their manufactured shape will be improved to allow for a more streamlined attachment to these animals.

While hatchlings were clearly attracted to the artificial light and spent more time in the tracking array than in ambient light conditions, they were not trapped indefinitely by the light and eventually continued their swim offshore. Hatchlings spent longer in the array on the first night of the experiment than the second. This was probably due to higher wind and larger waves that could have slowed their transit. Irrespective of these differences in weather conditions, we found similar outcomes on both nights with turtles consistently spending a longer time in the array during the light treatment than the ambient treatment. While symmetry of the placement of the light source between nights would have been preferred, our results do not appear to be related to the shorter distance from the release point to the light source on night 2 as the average time spent in the ambient treatment on night 2 was lower (12.46 ± 3.05 min) than in the light treatment on that night (17.75 ± 9.19 min). Importantly, the position of the light within an experimental unit (night) was constant.

One tagged turtle was recorded in the array for 28 h. Given the relatively rapid transit of all other individuals, we suggest that this turtle may have been eaten, with the tag then recording the presence of the predator and the tag within its stomach.

The change in turtle bearing between ambient and artificial light was greater on the second night of the experiment than the first. This result was due to differences in the position of the light in the array between nights. As mentioned previously, windy, shallow conditions on the first night of sampling combined with shallow water made it difficult for the boat (with the light) to anchor closer to shore.

As turtle hatchlings swim very quickly and the size of the array was relatively small, our tags had a fast transmission rate in order to obtain high resolution tracks. When faster transmitting tags are used on multiple animals in the same system there is an increased potential for signal collisions and, therefore, a reduction in the number of signals recorded by the array [32]. To reduce this risk, we released hatchlings into the array in pairs every 10 min. While more than two hatchlings could have been in the array at once without transmission collision, we took a very conservative approach to ensure the highest possible rates of data recovery from the tags.

Laboratory studies [10,33] and opportunistic observations in the field [4] have provided most of our knowledge of the in-water response of turtles to artificial light. Our experiment has provided some of the first data from *in situ*, at-sea experiments on wild hatchlings. We quantified the amount of time that hatchlings were attracted to lights and measured their swimming speed and orientation close to shore. We showed that current speed can influence hatchling movement, particularly when current direction is offshore and above speeds of approximately 32.5 cm s^−1^, but that artificial light appeared to be an overriding cue. Such information provides key behavioural insights that can be used to improve biophysical models of the dispersal of sea turtles. In the future it would be useful to examine the response of hatchlings to a range of different types and intensities of lights (cf. [2]) under different wind and wave conditions, and to more conclusively model the combined influence of current speed and direction on hatchling trajectories.

Our results show clear evidence that the behaviour of turtle hatchlings in the near shore is influenced by positive phototaxis, as is the case during the crawl from nest to sea. Furthermore, the transit of hatchlings across the near shore is delayed, probably increasing their risk of predation. These results have implications for both existing and future industrial developments near hatching beaches of marine turtles, and should be considered when planning and implementing such developments.

## Supplementary Material

Supplementary methods is a separate file which contains details on the pilot study work and supplementary figures related to the manuscript.

## Data accessibility

Data used in modelling are provided in the electronic supplementary material. Raw detection data and turtle tracks have been made available at the Australian Institute of Marine Science Data Centre at: http://data.aims.gov.au/metadataviewer/uuid/0503d5ff-d0f4-490d-ba6d-1c0d225b8996.
