# Supplementary material for: Artificial light on water attracts turtle hatchlings during their near shore transit
Source: R Soc Open Sci. 2016 May 18;3(5):160142. doi: 10.1098/rsos.160142 (PMC4892457; doi:10.1098/rsos.160142)
Supplement: Supplementary methods is a separate file which contains details on the pilot study work and supplementary figures related to the manuscript. [file rsos160142supp1.docx]

Supplementary material for:

Artificial light on water attracts turtle hatchlings during their nearshore transit

Thums et al.

Pilot study methods and results

The pilot study took place in February 2013 at Wobiri Beach (-21.82847, 114.06833), NorthWest Cape, northwest Western Australia. We first set up a miniature version of the array which consisted of 8 VR2W receivers (Vemco Ltd, Halifax, Canada) with co-located synch tags set up in two lines of three spaced 50 – 75 m apart and two reference tags in the intertidal zone. Each receiver was attached to a 1.5 m mooring line (4 mm propylene rope) that was held in position with a 100 mm subsurface float and a 3 kg weight. The synch tag was attached 0.5 m above the receiver with a zip tie and the height from the synch tag to the water surface was approximately 0.5 m. This test was left to run for 24 hours and at the end of the 24 hours a dummy turtle was towed around the array for 10 mins with an acoustic transmitter (V5 180 kHz coded acoustic transmitters, Vemco Ltd, Halifax, Canada, programmed with a delay of 5 to 10 seconds). We towed the dummy turtle at the surface to emulate the movement of green turtle hatchlings with the tag attached vertical to the long axis of the turtle.

Only five turtle positions were calculated during the test.  Receiver time synchronization was successful, with all receivers maintaining time synchronization with the system ~95% of the time.  System time synchronization requires that a synch tag be detected on at least two receivers simultaneously, and during the test we found that each synch tag transmission was detected 2.6 times on average.  Each turtle tag transmission, however, was only detected at 1.7 receivers on average.  In order to position the tag, the software requires simultaneous detections on at least three receivers, and unfortunately this was largely not the case.  Only 8% of the turtle tag transmissions were detected on three or more receivers.

From this test we determined that smaller receiver spacing was required. From advice from the manufacturer, we chose a conservative distance of 30 m and all 36 receivers were deployed in a 6 × 6 arrangement with the same procedures as above.

Hatchlings were instrumented with V5 transmitters which were glued to the turtle’s underside just prior to release using a small drop of Vetbond^TM^; a non-toxic, fast-acting adhesive used for veterinary procedures. The instrumented hatchlings were released into the array under natural conditions and with artificial lights present over two nights. All turtles were released into the array at the water’s edge, at the point on the shore in line with the middle receiver on the first line of the array. Lighting consisted of an industrial 400 watt metal halide light powered by a generator. The light was set up on an 8.25 m boat moored at the edge of the array with the light facing the beach and making a light spill near the boat. On the first night, 10 instrumented turtles were released into the array just after moon set with ambient light only. An hour later, the lights were switched on and another 10 instrumented turtles were released into the array and the lights then switched off 90 minutes later. On the following night the order was reversed. The following day the receivers in the array were retrieved and detection data downloaded and sent to Vemco for processing positions.

Only 40% of animal transmissions were detected on at least 3 receivers and this resulted in only 19 – 30% of animal transmissions resulting in a calculated position. On average only 2 positions were able to be calculated per turtle which was obviously not enough data points to constitute a track.   We hypothesized that because green turtle hatchlings swim at or very close to the surface and the tag was orientated parrallel to the longitudinal axis of the body that some of the acoustic signal was lost at the air-water interface. To test this hypothesis we attached the V5 tag to a dummy turtle in three different ways: 1. The tag attached horizontal to the body of the hatchling (as above), 2. vertical to the body and 3. hanging from a monofilament line. Of course this position was not possible on a real turtle but we contended that it was useful for establishing whether having the tag lower in the water would result in more transmissions received. The dummy turtle was towed around the array for 20 minutes.

The hanging deployment and vertical deployment was the most effective with around 80% of transmissions detected on at least 3 receivers and only 47% for our horizontal tag deployment (Table S1). Similarly around 70% of animal transmissions resulted in a calculated position with the hanging and vertical attachments compared with only 31% for the horizontal tag attachment (Table S1). Whereas three receivers have to hear each transmission for a successful position to be calculated, only 2.6 receivers heard each transmission on average for the horizontal attachment compared to 6 for the hanging and 4 for the vertical tag attachments. These results clearly show that at least for green turtle hatchlings, the tag must be positioned vertically with the transducer pointing down to successfully track these animals through an array.

1. Discussion

While the vertical transmitter attachment is likely to have more drag than the horizontal attachment, our pilot study showed that it was required to ensure successful tracking of the hatchlings through the array. Our testing suggests that the propensity of turtle hatchlings to swim at the surface means that some acoustic signal is lost at the sea surface rather than being directed downward towards the receivers on the benthos. This effect was reduced when the receiver was attached vertically. This propensity for surface swimming makes acoustic tracking of turtle hatchlings very challenging, compared to fish which are in the water column. The signal from the V5 transmitter is directed through the end of the transmitter which is not ideal for horizontal attachment to turtle hatchlings that swim at the surface, but it is currently the only acoustic transmitter small enough. Hopefully with the increase in the use of these transmitters on turtle hatchlings ([Scott et al., 2014](#_ENREF_1), [Thums et al., 2013](#_ENREF_2)) their manufactured shape will be improved to allow for a more streamlined attachment to these animals.

1. References

SCOTT, R., BIASTOCH, A., RODER, C., STIEBENS, V. A. & EIZAGUIRRE, C. 2014. *Nano-tags for neonates and ocean-mediated swimming behaviours linked to rapid dispersal of hatchling sea turtles*.

THUMS, M., WHITING, S. D., REISSER, J. W., PENDOLEY, K. L., PATTIARATCHI, C. B., HARCOURT, R. G., MCMAHON, C. R. & MEEKAN, M. G. 2013. Tracking sea turtle hatchlings - a pilot study using acoustic telemetry. *Journal of Experimental Marine Biology & Ecology,* 440**,** 156-163.


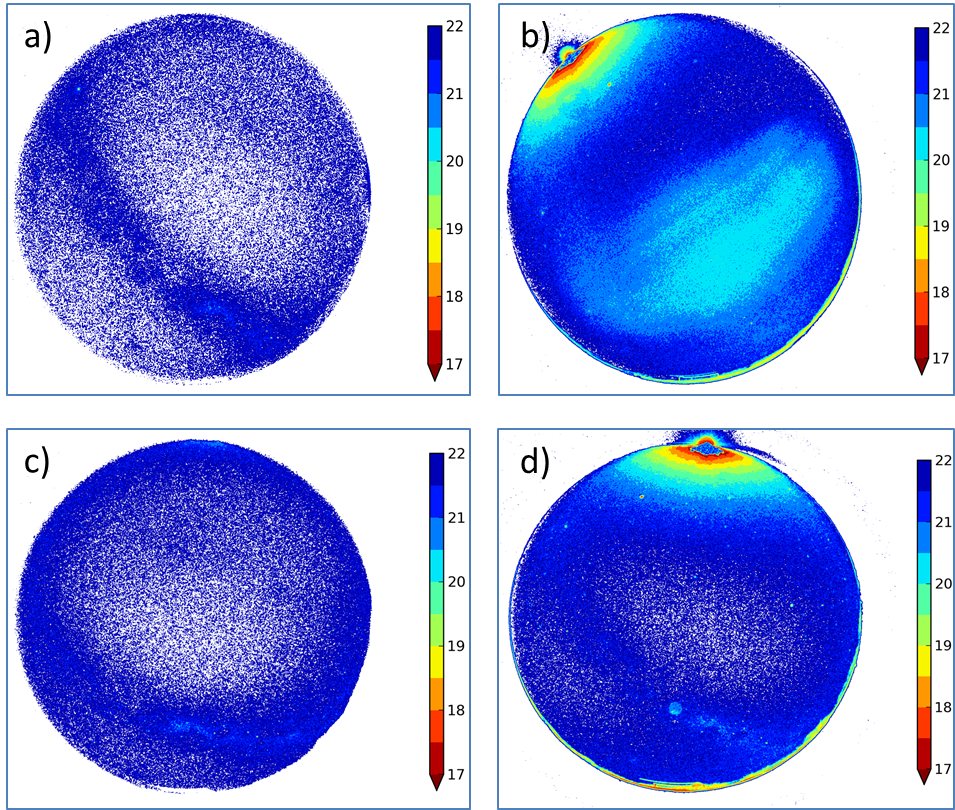


Figure S1. Isophote images showing the natural night sky on night 1 (a) and 2 (c) and when artificial light was switched on, on night 1 (b) and night 2 (d). Light intensity is presented on a logarithmic scale with larger values representing lower intensity light and darker sky, and smaller values represent higher intensity and brighter sky.


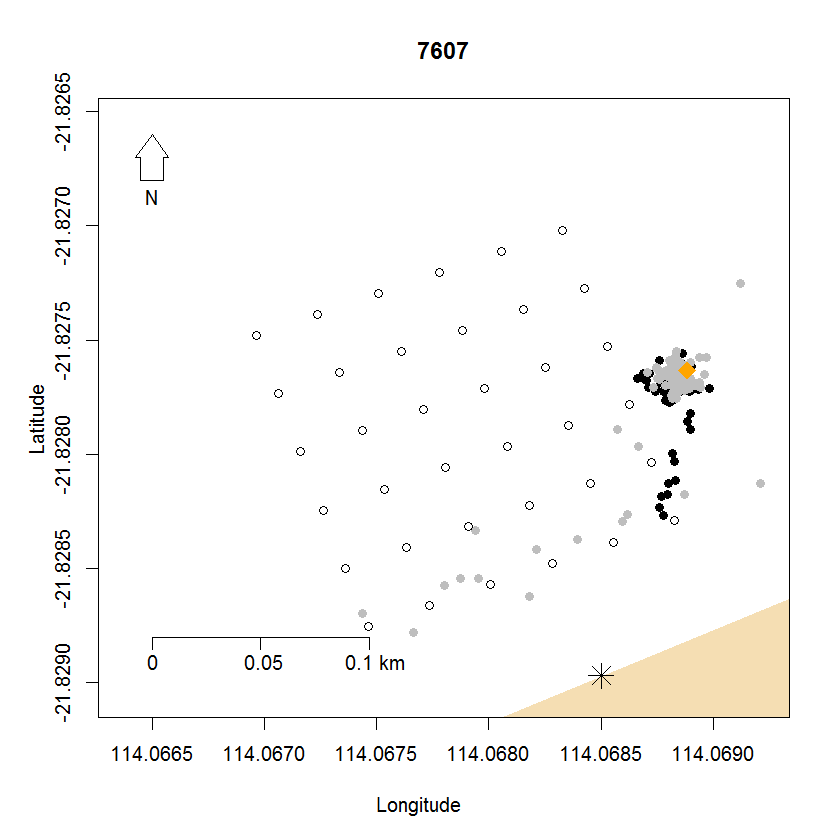


Figure S2. Positions of a turtle that was likely predated shown in filled circles. Black filled circles represent tracks received up to the maximum time that a hatchling spent in the array (34.5 mins) and thereafter filled circles are coloured grey. Open circles represent the positions of each of the receivers in the tracking array and the beach is shown in beige at the bottom right of the plot. The position of the light is indicated by the orange diamond.


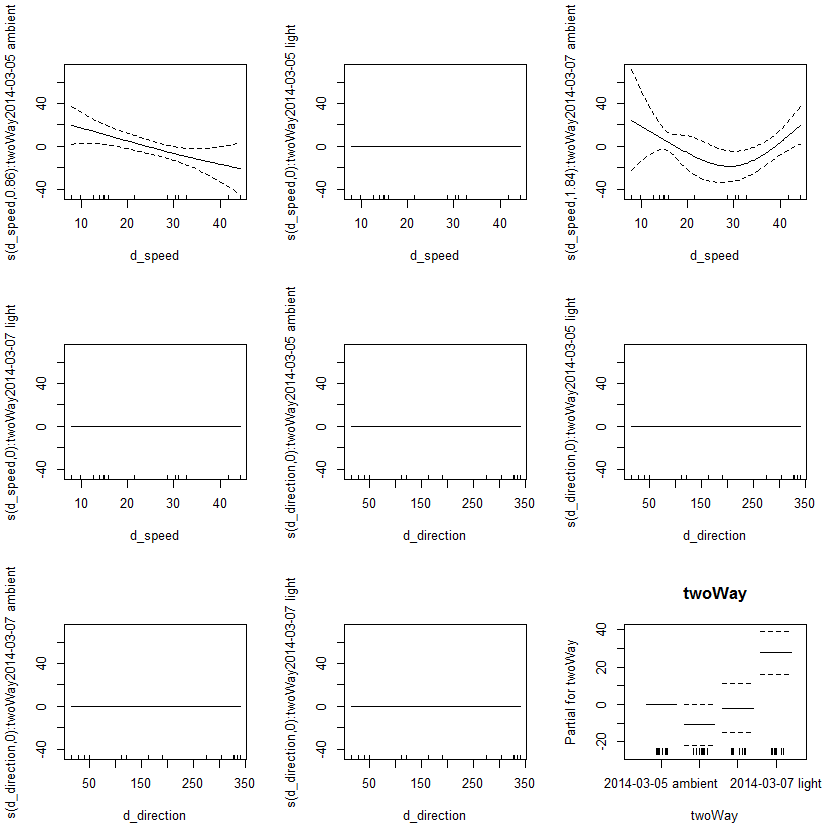


Figure S3. Fitted model for the three-way interaction between current speed (d_speed), light and night (two-way) and the three-way interaction between current direction (d_direction), light and night (two-way). This model had equal statistical support as the top model (within two AIC_c_ points) however was less parsimonious. It shows a relationship between turtle bearing and current speed only in the ambient treatments and no relationship with direction in any treatments. It also shows the partial plot (bottom right) for the two way interaction between treatment and night of the experiment.

Table S1. The results of testing using the three different attachment positions of the transmitter on the turtle (horizontal to the body, vertical to the body or hanging from the body on a line). Variables calculated for each test include number of detections, number of positions, % of transmissions detected on at least 3 receivers, % of transmissions resulting in a calculated position and the number of receivers hearing each transmission.

| **Variable** | **Horizontal** | **Vertical** | **Hanging** |
| --- | --- | --- | --- |
| Detections | 237 | 603 | 965 |
| Positions | 29 | 98 | 118 |
| % transmissions detected on at least 3 receivers | 47% | 80% | 81% |
| % transmissions resulting in a calculated position | 31% | 67% | 73% |
| Receivers hearing each transmission | 2.6 | 4.1 | 6.0 |

Table S2. Calculated statistics for each of the experimental nights. Note that bearing and speed could not be calculated for turtles 7624 and 7598 due to very low numbers of positions calculated (1 and 2 respectively). Number of current records refers to how many records were recorded by the current meter during the time the turtle was detected in the tracking array. Where more than one was recorded the current statistics were averaged. Where there were no current records, we took the closest measurement in time (time of current record).

| ID | Night | Start time | Treatment | Time spent (mins) | Turtle Bearing (degrees) | Turtle speed (cm s^-1^) | # current records | Time of current record | Current speed (cm s^-1^) | Current direction (degrees) |
| --- | --- | --- | --- | --- | --- | --- | --- | --- | --- | --- |
| 7627 | 1 | 2014-03-05 23:00:39 | ambient | 18.23 | -0.60 | 0.59 | 1 | 2014-03-05 23:06:00 | 14.9 | 111.24 |
| 7621 | 1 | 2014-03-05 23:00:51 | ambient | 19.12 | -21.42 | 0.56 | 1 | 2014-03-05 23:06:00 | 14.9 | 111.24 |
| 7623 | 1 | 2014-03-05 23:09:45 | ambient | 20.45 | -14.78 | 0.54 | 1 | 2014-03-05 23:24:00 | 30.37 | 120.3 |
| 7631 | 1 | 2014-03-05 23:10:04 | ambient | 20.90 | -20.31 | 0.44 | 1 | 2014-03-05 23:24:00 | 30.37 | 120.3 |
| 7617 | 1 | 2014-03-05 23:20:38 | ambient | 20.37 | -31.46 | 0.67 | 1 | 2014-03-05 23:24:00 | 30.37 | 120.3 |
| 7632 | 1 | 2014-03-05 23:22:09 | ambient | 19.67 | -41.07 | 0.50 | 1 | 2014-03-05 23:24:00 | 30.37 | 120.3 |
| 7616 | 1 | 2014-03-05 23:30:16 | ambient | 20.28 | 1.64 | 0.61 | 1 | 2014-03-05 23:42:00 | 14.71 | 14.65 |
| 7624 | 1 | 2014-03-05 23:33:45 | ambient | 7.30 |  |  | 0 | 2014-03-05 23:42:00 | 14.71 | 14.65 |
| 7620 | 1 | 2014-03-05 23:40:14 | ambient | 20.22 | -18.42 | 0.53 | 2 | 2014-03-05 23:51:00 | 30.99 | 27.3 |
| 7622 | 1 | 2014-03-05 23:40:38 | ambient | 25.20 | -43.49 | 0.52 | 2 | 2014-03-05 23:51:00 | 30.99 | 27.3 |
| Mean |  |  |  | 19.17 | -21.10 | 0.55 |  |  |  |  |
| SD |  |  |  | 4.56 | 15.78 | 0.07 |  |  |  |  |
| 7612 | 1 | 2014-03-06 00:00:51 | light | 21.90 | -7.82 | 0.51 | 1 | 2014-03-06 00:18:00 | 13.83 | -169.04 |
| 7633 | 1 | 2014-03-06 00:01:38 | light | 22.83 | -40.42 | 0.46 | 1 | 2014-03-06 00:18:00 | 13.83 | -169.04 |
| 7625 | 1 | 2014-03-06 00:10:37 | light | 23.05 | -28.26 | 0.42 | 1 | 2014-03-06 00:18:00 | 13.83 | -169.04 |
| 7618 | 1 | 2014-03-06 00:12:45 | light | 22.20 | -40.64 | 0.44 | 1 | 2014-03-06 00:18:00 | 13.83 | -169.04 |
| 7629 | 1 | 2014-03-06 00:20:20 | light | 21.93 | -38.32 | 0.58 | 1 | 2014-03-06 00:36:00 | 21.5 | -29.82 |
| 7619 | 1 | 2014-03-06 00:20:37 | light | 22.68 | -30.77 | 0.56 | 1 | 2014-03-06 00:36:00 | 21.5 | -29.82 |
| 7613 | 1 | 2014-03-06 00:31:09 | light | 20.78 | -37.32 | 0.51 | 1 | 2014-03-06 00:36:00 | 21.5 | -29.82 |
| 7628 | 1 | 2014-03-06 00:31:24 | light | 20.72 | -41.72 | 0.47 | 1 | 2014-03-06 00:36:00 | 21.5 | -29.82 |
| 7630 | 1 | 2014-03-06 00:41:19 | light | 25.48 | -40.33 | 0.39 | 1 | 2014-03-06 00:54:00 | 9.91 | 48.8 |
| 7626 | 1 | 2014-03-06 00:41:36 | light | 20.52 | -3.70 | 0.51 | 1 | 2014-03-06 00:54:00 | 9.91 | 48.8 |
| Mean |  |  |  | 22.21 | -30.93 | 0.48 |  |  |  |  |
| SD |  |  |  | 1.47 | 14.01 | 0.06 |  |  |  |  |
| 7609 | 2 | 2014-03-07 23:35:44 | light | 17.62 | 11.39 | 0.42 | 1 | 2014-03-07 23:42:00 | 44.28 | -19.9 |
| 7596 | 2 | 2014-03-07 23:36:17 | light | 34.53 | 12.99 | 0.44 | 2 | 2014-03-07 23:51:00 | 7.9 | -24.05 |
| 7597 | 2 | 2014-03-07 23:45:02 | light | 10.85 | 1.64 | 0.46 | 0 | 2014-03-07 23:42:00 | 44.28 | -19.9 |
| 7601 | 2 | 2014-03-07 23:45:29 | light | 17.17 | 10.38 | 0.48 | 1 | 2014-03-08 00:00:00 | 28.55 | 162.4 |
| 7600 | 2 | 2014-03-07 23:55:55 | light | 10.05 | -1.15 | 0.46 | 1 | 2014-03-08 00:00:00 | 28.55 | 162.4 |
| 7603 | 2 | 2014-03-08 00:06:08 | light | 24.67 | 11.54 | 0.45 | 1 | 2014-03-08 00:18:00 | 12.7 | 40.15 |
| 7615 | 2 | 2014-03-08 00:06:19 | light | 9.40 | 7.08 | 0.56 | 0 | 2014-03-08 00:18:00 | 12.7 | 40.15 |
| Mean |  |  |  | 17.75 | 7.70 | 0.47 |  |  |  |  |
| SD |  |  |  | 9.19 | 5.46 | 0.04 |  |  |  |  |
| 7611 | 2 | 2014-03-08 01:01:26 | ambient | 9.92 | 2.52 | 0.69 | 0 | 2014-03-08 01:12:00 | 41.89 | -32.86 |
| 7614 | 2 | 2014-03-08 01:01:42 | ambient | 11.98 | -25.17 | 0.51 | 1 | 2014-03-08 01:12:00 | 41.89 | -32.86 |
| 7610 | 2 | 2014-03-08 01:10:19 | ambient | 12.27 | -11.34 | 0.49 | 1 | 2014-03-08 01:12:00 | 41.89 | -32.86 |
| 7595 | 2 | 2014-03-08 01:11:08 | ambient | 16.32 | -7.03 | 0.41 | 1 | 2014-03-08 01:12:00 | 41.89 | -32.86 |
| 7594 | 2 | 2014-03-08 01:20:43 | ambient | 15.52 | -40.21 | 0.46 | 1 | 2014-03-08 01:30:00 | 32.61 | -85.59 |
| 7604 | 2 | 2014-03-08 01:23:48 | ambient | 9.85 | -48.18 | 0.40 | 1 | 2014-03-08 01:30:00 | 32.61 | -85.59 |
| 7599 | 2 | 2014-03-08 01:31:00 | ambient | 16.03 | -17.36 | 0.35 | 0 | 2014-03-08 01:48:00 | 15.84 | -55.11 |
| 7602 | 2 | 2014-03-08 01:32:02 | ambient | 12.45 | -35.20 | 0.44 | 0 | 2014-03-08 01:30:00 | 32.61 | -85.59 |
| 7605 | 2 | 2014-03-08 01:41:29 | ambient | 13.48 | -15.20 | 0.49 | 1 | 2014-03-08 01:48:00 | 15.84 | -55.11 |
| 7598 | 2 | 2014-03-08 01:41:52 | ambient | 6.80 |  |  | 1 | 2014-03-08 01:48:00 | 15.84 | -55.11 |
| Mean |  |  |  | 12.46 | -21.91 | 0.47 |  |  |  |  |
| SD |  |  |  | 3.05 | 16.61 | 0.10 |  |  |  |  |
